# Ligature of the Right Subclaclavian Artery

**Published:** 1864-02

**Authors:** P. F. Browne

**Affiliations:** Chimborazo, Division No. 1.


Art. IV.—*Ligature of the Right Subclavian Artery.* Reported by Surgeon P. F. Browne, Chimborazo, Division No. I.

Private J. B. Click, company "G," 5th Virginia cavalry, was wounded November 8th, 1863, at Brandy Station, by a minie ball, which entered the anterior fold of the right axilla about its middle, ranged through the axillary space, and was removed by counter-incision between the spinal column and the vertebral border of the scapula.

When admitted into the hospital, November 9th, the day after the receipt of his wound, there was no indication of any more serious injury than is usual in flesh wounds. All of his symptoms were favorable, and he rested easy till the fifth day, when he complained of very severe pain, extending from the shoulder to the tips of his fingers. This was accompanied with sleeplessness, a costive state of the bowels and great weakness. He continued suffering more or less in this way till December 3d. A small, hard and circumscribed tumor was then detected, for the first time, under the tendon of the pectoralis major. This tumor increased rapidly in volume.

On the 6th December fluctuation and also pulsation became evident, and, on auscultation, a double sound similar to the bellows' murmur of the heart. No thrill was perceptible either in the tumor or the radial artery. The symptoms were still too obscure to determine accurately its character; and opinion was very much divided. Some maintained strenuously that it was an abscess, from the very feeble pulsation and entire absence of all thrill, and also from the fact that pressure upon the subclavian over the first rib failed to diminish the size of the tumor. Others were disposed to regard it as an arterio-venous aneurism; and others again asserted that it was an extravasation of blood produced by ulceration of the coats of a vein.

On the 9th December all pulsation in the tumor and in the brachial and radial arteries ceased suddenly. The bellows' murmur also ceased. This was evidently due to great pressure upon the axillary artery by the greatly increased size of the tumor.

An exploring needle was now introduced, and a few drops of grumous blood escaped, but no trace of pus could be detected. The diagnosis being still doubtful, he was suffered to remain until the 15th. A trocar was then introduced, when, as before, dark blood only escaped. It was then decided to ligate the subclavian. The operation was performed on the 16th, by Assistant Surgeon J. C. Baylor, the artery being tied in the third part of its course.

After the application of the ligature, a consultation was held as to the propriety of opening the sac, which, by the great pressure it exerted over the axillary plexus of nerves was rapidly exhausting the patient. It was decided to lay it open by a free incision. This was accordingly done, and an immense clot exposed. On passing the finger into the clot, the artery spouted, and profuse hemorrhage ensued that threatened a speedy termination to life. The clot was quickly turned out and several attempts made to secure the bleeding vessel, but they proved ineffectual, and, as a last resort, the tampon was used. This hemorrhage was thus arrested, but the patient was left in an exceedingly feeble and prostrated condition. Stimulants were freely administered, but he sank rapidly, and died in eighteen hours after the operation. An autopsy held the next day revealed a lesion of the axillary artery just below the origin of the subscapular. The ligature was found firmly fastened around the subclavian, the inner and middle coats of which were divided. But as a week had elapsed since all pulsation had ceased in the tumor and the arteries below it before the operation was performed, time had been given for the establishment of anastomotic communication, and hence the hemorrhage.

[There are some points of great interest in this case: 1st, that so large a diffused aneurism should form, instead of having hemorrhage from the orifices of the wound; 2d, the point where the tumor showed itself; 3d, the patient did not die from *recurrent* hemorrhage, but the anastomoses, between the ligature and the point of ulceration, permitted the blood to flow directly from the artery.—ED]

